# Understanding Providers’ Offering and Patients’ Acceptance of HIV Screening in Emergency Departments: A Multilevel Analysis. ANRS 95008, Paris, France

**DOI:** 10.1371/journal.pone.0062686

**Published:** 2013-04-29

**Authors:** Kayigan Wilson d’Almeida, Dominique Pateron, Gérald Kierzek, Bertrand Renaud, Caroline Semaille, Pierre de Truchis, François Simon, Judith Leblanc, France Lert, Stéphane Le Vu, Anne-Claude Crémieux

**Affiliations:** 1 INSERM U1018, Centre de recherche en épidémiologie et santé des populations, Villejuif, France; 2 Université. Versailles Saint-Quentin, Faculté de Médecine Paris Île-de-France Ouest, EA 4499, Paris, France; 3 Service d’Accueil et de Traitement des Urgences, Hôtel-Dieu Cochin, AP–HP, Paris, France; 4 Département de Médecine Aigüe Spécialisée, Hôpital Raymond-Poincaré, AP–HP, Garches, France; 5 Institut de Veille Sanitaire, St-Maurice, France; 6 Service d’Accueil et de Traitement des Urgences, Hôpital Saint-Antoine, AP–HP, Paris, France; 7 Service d’Accueil et de Traitement des Urgences, Hôpital Henri Mondor, AP–HP, Créteil, France; 8 Service de Bactériologie–Virologie, Hôpital Saint-Louis, AP–HP, Paris, France; Fundacion Huesped, Argentina

## Abstract

**Objective:**

We assessed the EDs’ characteristics associated with the offer and acceptance rates of a nontargeted HIV rapid-test screening in 29 Emergency Departments (EDs) in the metropolitan Paris region (11.7 million inhabitants), where half of France’s new HIV cases are diagnosed annually.

**Methods:**

EDs nurses offered testing to all patients 18–64-year-old, able to provide consent, either with or without supplemental staff (hybrid staff model or indigenous staff model). The EDS’ characteristics collected included structural characteristics (location, type, size), daily workload (patients’ number and severity, length of stay in hours), staff’s participation (training, support to the intervention, leadership), type of week day (weekends vs weekdays) and time (in days). Associations between these variables and the staff model, the offer and acceptance rates were studied using multilevel modeling.

**Results:**

Indigenous staff model was more frequent in EDs with a lower daily patient flow and a higher staff support score to the intervention. In indigenous-model EDs, the offer rate was associated with the patient flow (OR = 0.838, 95% CI = 0.773–0.908), was lower during weekends (OR = 0.623, 95% CI = 0.581–0.667) and decreased over time (OR = 0.978, 95% CI = 0.975–0.981). Similar results were found in hybrid-model EDs. Acceptance was poorly associated with EDs characteristics in indigenous-model EDs while in hybrid-model EDs it was lower during weekends (OR = 0.713, 95% CI = 0.623–0.816) and increased after the first positive test (OR = 1.526, 95% CI = 1.142–2.038).

The EDs’ characteristics explained respectively 38.5% and 15% of the total variance in the offer rate across indigenous model-EDs and hybrid model-EDs vs 12% and 1% for the acceptance rate.

**Conclusion:**

Our findings suggest the need for taking into account EDs’ characteristics while considering the implementation of an ED-based HIV screening program. Strategies allowing the optimization of human resources’ utilization such as HIV targeted screening in the EDs might be privileged.

## Introduction

Emergency Departments (EDs) provide care for high proportions of the populations in developed countries [1], [2]. In France, nearly a quarter of the population visits EDs each year [2]. Therefore, in addition to their primary acute-care role, EDs appear as potential places to provide preventive health care [3]–[6]. Because screening is a component of secondary disease prevention, many attempts have been made to implement ED screening programs for a range of conditions including depression [7], alcohol abuse [8], smoking [9], intimate partner violence [10], diabetes [11], hypertension [12] and more recently HIV infection in the USA [13].

In 2006, CDC published recommendations for HIV screening in all EDs [13].^.^Since then, the number of EDs conducting HIV screening has grown. Yet, only 8% of the EDs in the US report universal HIV screening [14]. The paucity of evidence regarding the benefit of HIV nontargeted screening as a public health prevention strategy [15], [16], or regarding the best approach to use while conducting such a screening [17],combined with the lack of funding might partly explain why HIV screening is not widely available in EDs [18], [19]. In addition, in the settings where the implementation of HIV ED-based screening have been attempted, numerous barriers have been reported, including time constraints, inadequate resources, concerns regarding workloads or provision of follow up care [20]. Finally, HIV screening raises specific issues and legal concerns, such as the need for HIV screening programs to comply with HIV-test regulations [21] or the fact that some clinicians feel uncomfortable offering HIV testing and disclosing positive results [22].

Among the few EDs actually conducting HIV screening, a cross-site comparison of HIV screening programs in 6 US emergency departments found that structures and processes varied a lot among EDs, most sites using supplemental staff for testing [18]. Studies comparing the outcomes of ED-based screening programs in terms of offer rate, testing rate and acceptance rate suggested that those outcomes were better in models using a dedicated staff in comparison with approaches relying on ED staff only [23], [24]. However, in times of limited resources, it seems improbable that specific resources for HIV screening will be allocated to EDS. Therefore it could be useful for public health authorities and ED’s managers to better understand which EDs’ characteristics are associated with the probability of implementing HIV screening with an operational model rather than another one, and the EDs’ characteristics associated with the best ED-based HIV screening outcomes.

During the 2009–2010 period, we performed a study in 29 EDs [15] to evaluate the impact of nontargeted HIV screening in the Paris metropolitan region, which accounts for almost half of the HIV cases in France. In half of the 29 EDs, the screening program relied as initially expected on the EDs teams alone while in the other half, supplemental staff was needed to achieve the program. Using data collected during this study, our objectives were first to determine which ED’s characteristics (structural characteristics, staffs attitudes regarding HIV screening, daily patient flow) were associated with the implementation of an ED-based nontargeted HIV rapid-test (HIV-RT) screening without any supplemental staff and second to assess the ED’s characteristics associated with the offer and acceptance rates of HIV-RT screening within each operational model.

## Methods

This study was approved by Île-de-France XI Committee for Patient Protection (no. 08053, 9 October 2008) and by the ‘Commission Nationale de l’Informatique et des Libertés’ (French data protection authority). According to the protocol approved by this committee, a written informed consent was obtained for each participant.

### Sample and Study Design

From May 2009 to September 2010, we conducted an interventional study in 29 EDs located in the Paris metropolitan region (12 million inhabitants), which harbors 44% of the new HIV diagnoses in France [25]. In each ED, the intervention lasted 6 consecutive weeks, randomly allocated, during which HIV RTs (OraQuick Advance Rapid HIV-1/2 Antibody Test, Orasure Technologies Inc., Bethlehem, Pennsylvania) were performed on a 24-hour basis. During that period eligible patients were asked if they accepted to participate in this research, by undergoing free-of-charge HIV-RT (opt-in approach). Exclusion criteria were: self-reported HIV infection; inability to provide consent because of neuropsychiatric disorders, substance abuse, language barrier or being under arrest; unstable medical illness or consulting for HIV-postexposure prophylaxis. The rationale and interventions of this study as well as the characteristics of tested patients have been described in detail [15]. A research assistant (RA) was present at every site for 8 hours a day to monitor the study and remind the ED staff to offer HIV-RT to all eligible patients. After a week of observation, a debriefing session was systematically conducted in each site to determine whether the ED staff could achieve the intervention alone as outlined in the study protocol or if they needed support from the RA. In the latter case, the RA assisted or replaced the ED staff at various steps in the process: informing patients, obtaining consent, performing the test and disclosing negative test results. Subsequently, the 29 EDs participating in the study were classified in two staffing models: the indigenous staff model where the protocol could rely on the existing staff only and the hybrid staff model in which the RA had to provide support to the staff.

### Setting

The EDs recruited for this study participate in the French national acute syndrome-surveillance network (OSCOUR) [26]. This network was created to be an early warning system for community-wide illnesses and outbreaks. It is composed of 245 adult and pediatric emergency departments distributed all over the country, among which 31 are located in Paris region and account for 60% of all patients visiting EDs in the region [27]. Those EDs reflect the diversity of the French ED settings (academic, nonacademic, private, public departments). Each ED that participates in this network, daily transfers standardized information directly from the medical file of each patient into a centralized database. The data recorded in OSCOUR include demographics (age, sex), medical information (ICD10 diagnosis, severity score, reason for emergency admission), patient outcomes (transfer, admission, discharge) and real-time ED occupancy. The OSCOUR network investigators were approached before the beginning of the present study and accepted to provide extractions of their database for each ED’s study period.

### Data Collected

Prior to and during the intervention, we collected data from each ED at two levels: the ED-level and the daily activity-level. ED-level data included the ED structural characteristics and data related to the intervention including staff attitudes and levels of training. The day-level data included data related to the ED’s workload, which was mainly indicated by the patient flow, the proportion of seriously ill patients and the mean length of ED stay; the latter reflected the ability of EDs to manage their patient flow.

#### ED-level explanatory variables

The EDs were categorized by their academic or nonacademic status, location within or outside of Paris, ED/hospital bed capacity and average staffing levels (e.g., number of physicians, nurses or nursing assistants per 100 patients). The annual census was used as a proxy of the EDs’ size (<20000: small ED, 20000–35000: medium-low ED, 35000–50000: medium-high ED and >50000: large ED).

The teams’ support prior to the intervention was assessed by the study investigators after the preliminary meetings with the staff and the training sessions. We define a priori 5 criteria to evaluate this support: (1) the attendance of chief nurses and head of departments to the preliminary meetings, (2) the easy scheduling of training sessions (3) teams showing interest in the study through questions during the preliminary meetings and training sessions, (4) teams showing belief in the rationale of the study through positive attitudes and commentaries during preliminary meetings and training sessions, (5) teams spontaneously proposing possible processes to achieve the intervention in the ED. At the end of the sessions, each study investigator gave a mark between 1 (weak support) and 5 (full support) to the staffs based on their interest and belief in the rationale of the study. The support score was obtained by calculating the mean mark. We also documented the presence of a leader defined as a nurse or a physician who held a leadership position in the department and whose role was to promote the intervention and stimulate the staff throughout the six-week period. Data related to the staff training were collected. All the nurses were invited to participate in the training sessions, which were organized by the chief nurses. No specific sessions were planned for the physicians, who were allowed to participate in the nurses’ sessions if they desired to. Therefore, we reported the level of training for the nurses as a percentage of attendance to training sessions, whereas we reported the physicians’ participation in the training as a simple dichotomous variable (at least one physician or none).

#### Daily activity-level explanatory variables

The daily patient flow, the mean length of stay in hours and the proportion of seriously ill patients (emergency severity score <3, corresponding to patients with unstable medical illness in the triage scale currently used in French EDs) [28] were extracted from the OSCOUR database. To account for the daily variation in patient flow within each site and its variability across sites, we considered each site’s patient flow as a categorical variable using quartiles of distribution. We also separately analyzed weekend vs. weekdays. Finally, we hypothesized that the team attitudes toward the intervention might change during the 6-week intervention or after the occurrence of the first positive rapid test because it is a rare and serious event. Therefore, we included the time post the intervention beginning as a potential explanatory variable and dichotomized the study into two periods: either preceding or following the first positive HIV-RT.

### Statistical Analysis

First, we analyzed the association between the EDs’ characteristics and the implementation of the HIV screening program without supplemental staff. Then we assessed the factors associated with the HIV test offering (i.e. the proportion of HIV tests that were offered to eligible patients) and acceptance (i.e. the proportion of tests that were accepted when proposed). The number of tests performed was very close to the number of tests accepted so to avoid redundancy, we focused on the latter in the analyses.

The proportions were compared using a chi-squared test or Fisher’s exact test and means were compared using Student’s t-test. To account for the hierarchical structure of our data, in which days of activity were nested within the EDs, we used a multilevel analysis that is recommended for testing the effects of group-level variables [29] to divide the total variance in offering or acceptance into the variance at the ED-level and the variance at the daily activity-level. We performed a multilevel logistic regression analysis using the SAS PROC GLIMMIX procedure (version 9.3; SAS Institute, Inc., Cary, NC) to analyze between and within EDs’ variability in the offering and patient acceptance of HIV-RT. To calculate the variance partition coefficient (VPC) which indicates the proportion of variance accounted for by the ED level, we first modeled the “empty” model using a random intercept and no explanatory variables. Then we proceeded with the “full” model, including the effects of ED’s characteristics. Measures of association were provided as multivariate odds ratios (OR). We assessed the proportional reduction in variance after adjusting for the ED’s characteristics, which were significant in the bivariate analyses. We anticipated that the factors associated with the offering and acceptance of HIV-RT would be different depending on whether an additional staff (RA) was needed or not. Therefore, we conducted stratified analyses based on that dichotomization. We applied a significance level of 5% to all of the two-sided statistical tests.

## Results

**Figure 1:**
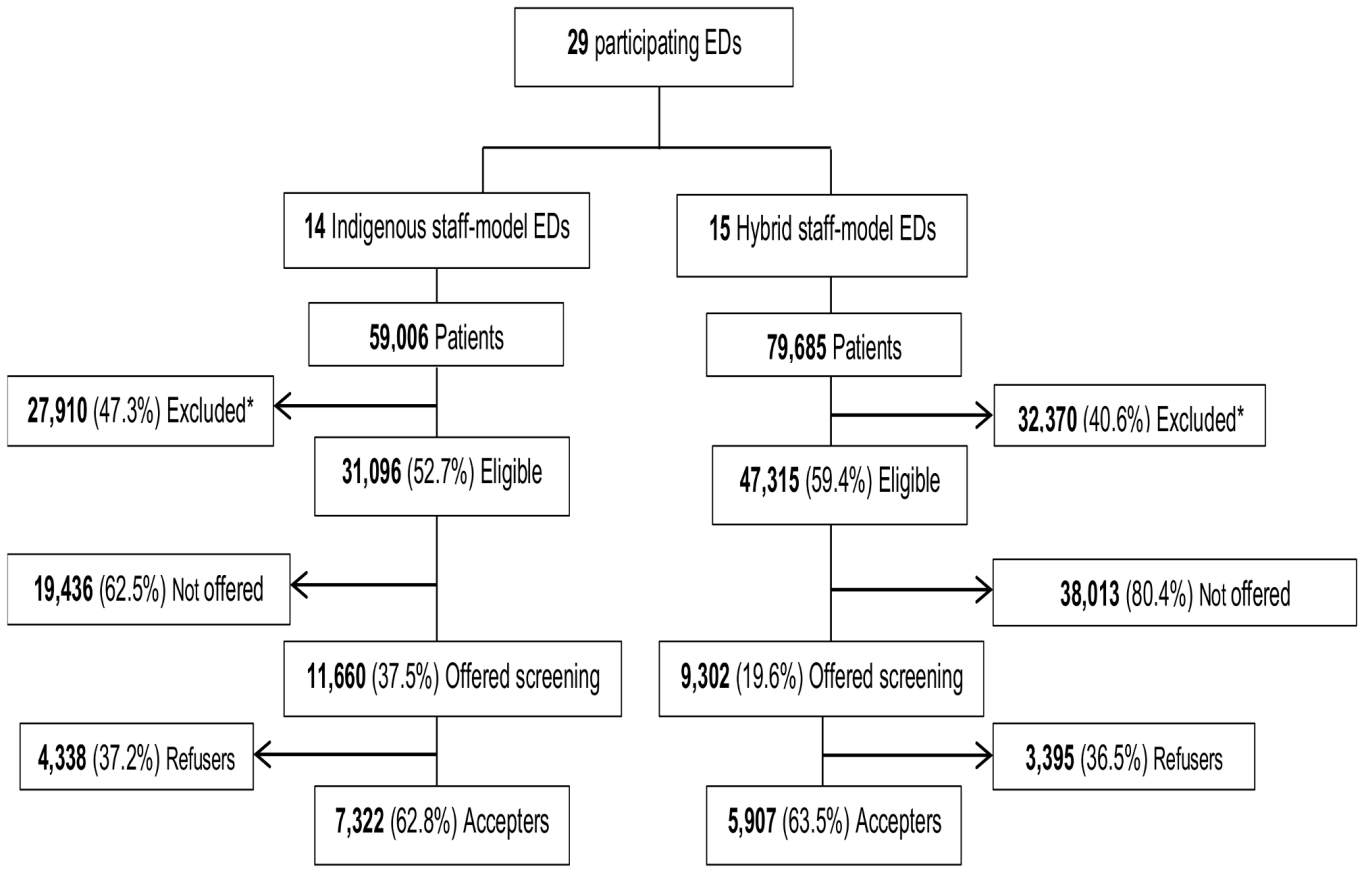
Patient flow diagram. *Exclusion criteria were the following: age <18 years or age >65 years (51%), serious/unstable illness (15%), inability to provide consent (31%), known HIV seropositivity (2%).

Out of 138,691 patients that visited EDs during the study period, 78,411 (56.5%) were eligible for HIV screening. Among these patients, 20,962 were offered HIV-RT (offer rate = 26.7%, varying from 11% to 88%) and 13,229 accepted (acceptance rate = 63.1%, varying from 44% to 87%). The indigenous staff model was used in 14/29 EDs and the hybrid staff model was used in the other 15 EDs. Acceptance was similar (63%) in both groups of EDs whereas the offer rate was higher for the indigenous staff model (37% vs. 20%, P<.05) (Figure 1).The characteristics of the EDs sample are shown in table 1.

**Table 1:** Sample’s characteristics.

	Whole sample	Indigenous staff model-EDs	hybrid staff model-EDs
	(n = 29)	(n = 14)	(n = 15)
**ED CHARACTERISTICS**			
**Number of beds in the hospital**	482.8	**430.9**	**532.6** *
**Number of beds in the ED**	11.8	**10.6**	**12.9** *
**Number of physicians/100 patients**	6.5	**6.9**	**6.1** *
**Mean number of patient/day (IQR)**	114.6 (71–147)	**99.3 (56–134)**	**129.2 (94–157)** *
**Mean length of stay (hours)**	4.3	**3.6**	**4.9** *
**Proportion of seriously ill patients**	17	**21.6**	**12.7** *
**Hospital type** (n, %)			
academic	14 (48.3%)	**3 (21.4%)**	**11 (73.3%)** *
Non academic	15 (51.7%)	**11 (78.6%)**	**4 (26.7%)**
**Location (**n,%)			
Out of Paris city	22 (75.9%)	11 (78.6%)	11 (73.3%)
In Paris city	7 (24.1%)	3 (21.4%)	4 (26.7%)
**ED CHARACTERISTICS RELATED**			
**TO THE INTERVENTION**			
**Medical staff training** (n, %)			
No	14 (48.3%)	5 (35.7%)	9 (60%)
At least one physician	15 (51.7%)	9 (64.3%)	6 (40%)
**Proportion of nurses trained**	67%	73%	62%
**Mean support score**	3.55	**4.14**	**3.00** *
**Identification of a leader** (n, %)			
No	13 (44.8%)	4 (28.6%)	9 (60%)
Yes	16 (55.2%)	10 (71.4%)	6 (40%)

* p<0.05.

### Factors Associated with the Implementation of the HIV Screening Program without Supplemental Staff (Indigenous Staff Model) (Table 1)

The ED structural variables associated with the staffing model were the hospital type, the bed capacity and the staffing levels. Indigenous staff model-EDs belonged more frequently to nonacademic hospitals (78.6% vs. 26.7%), had a lower bed capacity (10.6 vs. 12.9 beds) and employed a higher number of physicians (6.9 vs. 6.1 physicians/100 patients). The daily workload variables associated with the staffing models were the patient flow, the mean length of stay and the proportion of seriously ill patients. Compared to hybrid staff model-EDs, the EDs using the indigenous staff model exhibited a lower daily patient flow (99.3 vs. 129.2), a shorter length of stay per patient (3.6 vs. 4.9 hours) and a higher proportion of seriously ill patients (21.6% vs. 12.7%). The mean team-support score prior to the intervention was higher for the indigenous staff model-EDs (4.1 vs. 3.04).

### Factors Associated with the Offering and Acceptance of HIV-RT within Indigenous Staff Model-EDs (Figure 2)

In the indigenous staff model-EDs, multivariate analyses showed that the offer rate decreased when the number of visits per day reached the 4th quartile (0.838 [0.773–0.908]) and when the mean length of stay per patient increased (0.885 [0.854–0.917]). The offer rate also decreased over time (0.978 [0.975–0.981]) and was lower on weekends (0.623 [0.581–0.667]). The offer rate increased with the level of staff support to the intervention (1.81 [1.152–2.851]). In these EDs, the only variable associated with acceptance was the size of the ED. The acceptance rate was lower in small (0.241 [0.135–0.427]), medium low (0.184 [0.091–0.371]) and medium high EDs (0.283 [0.154–0.518]) vs large EDs.

**Figure 2:**
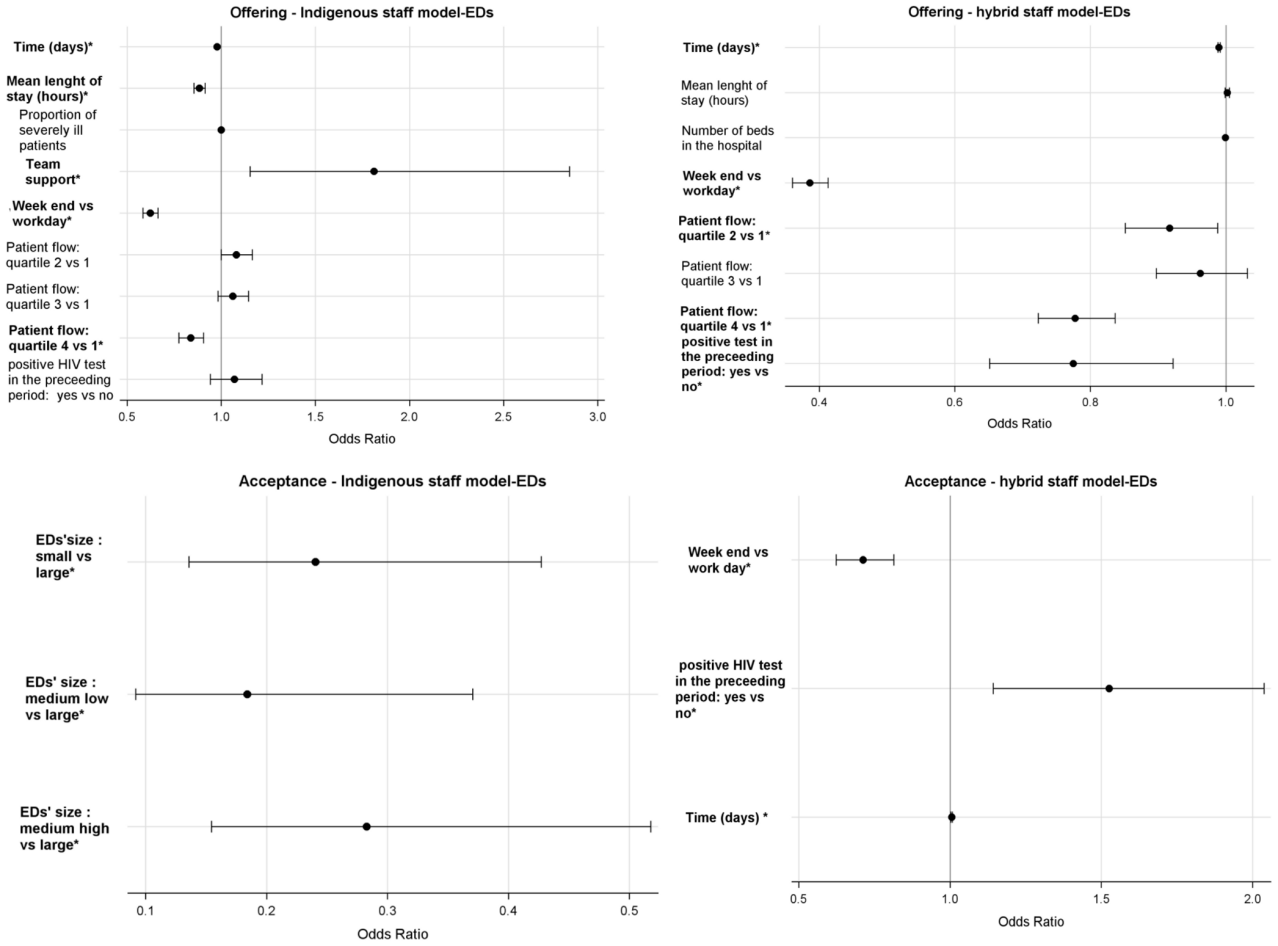
EDs’ characteristics associated with the offering and acceptance of HIV-RT in the EDs (multivariate analysis^a^). ^a^Team support, proportion of seriously ill patients, length of stay, time and number of beds in the hospital are continuous variables. All the others are categorical variables *P<.05.

### Factors Associated with the Offering and Acceptance of HIV-RT within Hybrid Staff-EDs (Figure 2)

Consistently with what was observed in the indigenous staff model, in the hybrid staff model, the offer rate was lower on weekends (0.386 [0.360–0.414]), decreased over time (0.990 [0.988–0.992]) and when the number of visits per day reached the 4th quartile (0.777 [0.722–0.837]). The offer rate also decreased after the occurrence of the first positive rapid test (0.775 [0.651–0.922]). In these EDs, the acceptance rate increased with time (1.005 [1.001–1.009]), was lower during the weekends (0.713 [0.623–0.816]) and increased after the occurrence of the first positive HIV-RT (1.526 [1.142–2.038]).

### Variance Partitioning

Based on the variance partition coefficient, table 2 shows which part of the variance in HIV-RT test offering and patients’ acceptance is attributable to differences across EDs in the two models. After controlling for EDs’ characteristics, the variance in test offering that was attributable to differences between EDs decreased from 19.5% to 12% in the indigenous staff model and from 6 to 5.1% in the hybrid staff model. Thus, the set of EDs’ characteristics introduced in the models explained 38.5% of the variance in offering at the ED-level in indigenous staff model-EDs and 15% of the variance in offering at the ED-level in hybrid staff model-EDs. In parallel, the EDs’ characteristics explained 12% of the variance in acceptance at the ED-level in indigenous staff model- EDs and 1% of the variance in acceptance at the ED-level in hybrid staff model-EDs.

**Table 2:** Components of the offering/acceptance variance and impact of entering EDs’ characteristics in the regression analysis.

	Indigenous staff model-EDs		Hybrid staff model-EDs	
Outcome	ED level variance	VPC*	ED level variance	VPC*
offering (empty model)	0.7985	19.5%	0.2256	6%
offering (after adjustment)	0.4534	12%	0.1790	5.1%
Acceptance (empty model)	0.3478	9%	0.1523	4.42%
Acceptance (after adjustment)	0.2813	7.9%	0.1506	4.37%

* The variance partition coefficient (VPC) indicates the proportion of the total variance (the sum of 1st level (days) and 2nd level (EDs) variances) that it is accounted for by the 2nd level variance. The VPC was calculated using the equation VPC_h_ = σ^2^_h_/(σ^2^_h_ +3.29), where σ^2^_h_ represents the ED level variance (Snijders, T. and Bosker, R. [1999]. Multilevel Analysis. Sage).

## Discussion

The present study was conducted in a large and diverse sample of Paris Region’s EDs.

In our protocol, the EDs existing staff was initially expected to offer testing, perform the rapid tests and disclose the results without any support from the RA. In practice, the intervention entirely relied on EDs teams in only half of our sample while in the other half support from the RA was needed for some part of the testing procedures. That situation provided us with the opportunity of analyzing the EDs’ characteristics associated to the staffs’ autonomy regarding the intervention, which was our first objective in the present study. Our analysis of the factors associated with the staffing model showed that the structural characteristics and workload parameters of the EDs were strongly associated with the staffs’ autonomy regarding the implementation of such an intervention. The largest departments, which were located in academic settings and exhibited higher patient flows, lower number of physician/100 patients and longer lengths of stay, were less inclined to complete the whole process from the offering of the test to the disclosure of the results. Such findings are concordant with the fact that concerns about additional workload are one of the main barriers to the implementation of universal screening in US EDs as recommended by the CDC [21]. Therefore, considering existing EDs constraints, HIV testing strategies focusing on the patients most at risk should be privileged in order to optimize human resources utilization [30]. Our study also underlined the staff motivation as an important driver to implement HIV screening: the more interested the ED staff members were in the rationale of the study prior to the intervention, the more frequently these EDs achieved the intervention without using additional research personnel.

The second goal of our study was to explore the factors associated with the offering and acceptance of an ED-based HIV nontargeted screening program within each staffing model.

The main determinant of HIV-RT offering was the patient flow. In both groups of EDs, HIV-RT offering decreased when the patient volume increased, but this association was not linear; furthermore, the greatest decrease in HIV-RT offering occurred when the patient flow reached a threshold (4^th^ quartile) that corresponded to the peak periods of overcrowding. HIV-RT offering was lower during weekends. Indeed, the ED utilization is higher [31] and the staffing levels are lower during weekends compared with the weekdays. Taken together those results confirm that the workload is a major barrier to the implementation of ED-based HIV screening as it is significantly associated not only to the autonomy of the EDs’teams regarding the intervention but also to the offer rate within each operational model. Similarly, the level of support appeared as essential both for the implementation of the program without supplemental staff and for the optimal offering of HIV screening in the EDs where the teams offered and performed the tests without the RA. Another interesting finding is that, in both groups of EDs, HIV-RT offering decreased with time. Similar result has been described in a study performed during a longer period in a smaller sample of EDs of the same region [32]. It might be explained by the fact that it is difficult to maintain a sustain mobilization for an activity which increases the workload for EDs’teams.

In hybrid staff model-EDs, the occurrence of the first positive rapid test was associated with a decrease in further HIV-RT offering. Actually, a positive result was an uncommon event (38 positive RT/12754 tests, of which, 18 were determined to be new diagnoses after interviewing the patient) which might have been experienced as stressful and might have affected the staffs or RA’s attitudes toward the HIV-RT offering. This observation merits further investigation as we did not have the opportunity to measure change in attitudes in the present study.

Few of the assessed variables were associated with the patients’ acceptance of an HIV-RT in the EDs. In indigenous staff model-EDs, the only variable associated with the patients’ acceptance was the ED’s size with a lower acceptance rate in smaller EDs. In hybrid staff model-EDs, the patients’ acceptance increased with time and after the occurrence of the first positive test. Additional studies are necessary to clarify these associations.

Finally, the multilevel statistical analysis allowed us to assess which part of the variance in HIV-RT offering and acceptance was attributable to differences between the EDs. As expected, the ED-related factors explained a larger proportion of the variance between indigenous staff model-EDs than between hybrid staff model ones. Indeed, in the latter group, additional research staff was available to compensate for the teams’ difficulties; thus, the team’s performance might have been less affected by the work context. The variance in HIV-RT acceptance that was attributable to differences between the EDs was smaller than for HIV-RT offering. As determined by previous studies, consent to be tested is associated with individual factors such as the gender, HIV risk behavior, history of previous HIV screening and perceived risk of HIV infection [33]–[36]. In the present study, patients were found to be less likely to accept the test if they perceived themselves to be at low risk of contracting HIV or if they had been previously tested [15]. In the same idea, contrary to what was found by Hutchinson et al. [37], in our study the acceptance rate was similar in both models suggesting that once the test is offered, acceptance appears to be rather patient-related than staff-related.

Our study has several limitations. First, the intervention lasted only 6 weeks in each ED. The determinants of the offering and acceptance of ED-based HIV screening might not have been the same over a longer period or if HIV testing had been integrated into the EDs’ routines. Second, these results apply to nontargeted HIV screening and should not be extrapolated to targeted HIV screening. Third, we were not able to include the patients’ individual characteristics in the dataset used for the multilevel analyses because patients who were not proposed or who were not eligible were not asked to complete the study questionnaire. Finally, we did not compare the outcomes of the two operational models in this analysis. Indeed, it was not the first goal of our study and this point which has been previously addressed [23], [24] would have required a randomization in order to control for biases. The major strength of this study is that to our knowledge, it is the first to assess the EDs’ characteristics associated with the implementation and outcomes of an ED-based HIV screening intervention in a large sample of EDs at a region-wide scale. The use of the OSCOUR database provided a wide range of harmonized variables which roles were assessed through a multilevel analysis that considered the hierarchical structure of our data.

In summary, patients’ acceptance of HIV screening is high in all EDs, confirming the acceptability of provider-initiated testing in the French general population [38]: when nurses are willing and able to propose HIV testing, the offering is introduced in a manner that allows patients to accept or decline it, based on their individual perceptions, behaviors and readiness. Supplemental staff is needed to integrate routine HIV screening in large hospitals with overcrowded EDs. Nevertheless, whatever the operational model, the offer rate declines when the patient flow reaches its highest level, which underlines the difficulty of implementing HIV routine screening in emergency departments. Taken together, these findings suggest the need for strategies allowing the optimization of human resources’ utilization such as ED-based HIV targeted screening, which should be further evaluated.
